# Modelling Neurotropic Flavivirus Infection in Human Induced Pluripotent Stem Cell-Derived Systems

**DOI:** 10.3390/ijms20215404

**Published:** 2019-10-30

**Authors:** Giovanna Desole, Alessandro Sinigaglia, Silvia Riccetti, Giulia Masi, Monia Pacenti, Marta Trevisan, Luisa Barzon

**Affiliations:** 1 Department of Molecular Medicine, University of Padova, 35121 Padova, Italy; giovanna.desole88@gmail.com (G.D.); alessandro.sinigaglia@unipd.it (A.S.); silvia.riccetti@studenti.unipd.it (S.R.); giulia.masi@unipd.it (G.M.); 2 Microbiology and Virology Unit, Padova University Hospital, 35128 Padova, Italy; monia.pacenti@aopd.veneto.it

**Keywords:** Zika virus, West Nile virus, dengue virus, flavivirus, neural stem cell, neuron, induced pluripotent stem cell, innate immune response, viral replication, infection, apoptosis

## Abstract

Generation of human induced pluripotent stem cells (hiPSCs) and their differentiation into a variety of cells and organoids have allowed setting up versatile, non-invasive, ethically sustainable, and patient-specific models for the investigation of the mechanisms of human diseases, including viral infections and host–pathogen interactions. In this study, we investigated and compared the infectivity and replication kinetics in hiPSCs, hiPSC-derived neural stem cells (NSCs) and undifferentiated neurons, and the effect of viral infection on host innate antiviral responses of representative flaviviruses associated with diverse neurological diseases, i.e., Zika virus (ZIKV), West Nile virus (WNV), and dengue virus (DENV). In addition, we exploited hiPSCs to model ZIKV infection in the embryo and during neurogenesis. The results of this study confirmed the tropism of ZIKV for NSCs, but showed that WNV replicated in these cells with much higher efficiency than ZIKV and DENV, inducing massive cell death. Although with lower efficiency, all flaviviruses could also infect pluripotent stem cells and neurons, inducing similar patterns of antiviral innate immune response gene expression. While showing the usefulness of hiPSC-based infection models, these findings suggest that additional virus-specific mechanisms, beyond neural tropism, are responsible for the peculiarities of disease phenotype in humans.

## 1. Introduction

The *Flavivirus* genus of the family *Flaviviridae* includes several arthropod-borne human pathogens that may cause a spectrum of clinical syndromes ranging from a mild febrile illness to severe hemorrhagic fever or neuroinvasive disease. Neurotropic flaviviruses, such as West Nile virus (WNV), Japanese encephalitis virus (JEV), and tick-borne encephalitis virus (TBEV), are maintained in nature in transmission cycles involving mosquito (mainly *Culex* spp.) or tick (mainly *Ixodes* spp.) vectors and mammalian or bird amplification hosts, with humans serving as dead-end incidental hosts. Most human infections are asymptomatic or characterized by flu-like illness, while a severe and potentially fatal neuroinvasive disease occurs in a minority of cases, mostly immunocompromised and elderly individuals [1].

Zika virus (ZIKV) has recently drawn attention due to the large outbreaks that occurred in Pacific Ocean countries and the Americas. Like other flaviviruses, ZIKV generally causes asymptomatic infections or a mild febrile illness [2]. Infection during pregnancy, especially during the first trimester, causes fetal microcephaly and other developmental anomalies, defined as congenital Zika virus syndrome [3]. Guillain–Barré syndrome and meningoencephalitis are rare complications of ZIKV infection in adult individuals. ZIKV is transmitted between humans through the bite of infected *Ae. aegypti* and other *Aedes* species mosquitoes, with humans serving as amplifying hosts. Unique among flaviviruses, ZIKV can persist in immune-privileged sites like the testis and be sexually transmitted [4,5].

In addition to these well-known neurotropic flaviviruses, there has been an increasing number of reported cases of neurological complications caused by viruses, such as dengue virus (DENV) and yellow fever virus (YFV), which usually are associated with hemorrhagic disease [6,7].

One of the central questions in the search for therapeutic targets for the treatment of flaviviral neurological syndromes is whether these diseases are induced by virus-mediated cytolysis, immune-pathological responses to infection, or a combination of both [8,9]. Animal models have permitted temporal analysis of the progressive dissemination of virus infection within the CNS and identification of cells targeted by viral infection, have revealed important details regarding host factors that restrict viral replication and the innate and adaptive immune responses to viral infections, and have been fundamental for vaccine development [10,11]. Breakthroughs on cell reprogramming and generation of human induced pluripotent stem cells (hiPSCs) have revolutionized approaches to the study of human diseases. iPSCs are generated directly from adult differentiated cells that are reprogrammed back into an embryonic-like pluripotent state by introducing genes important for maintaining the essential properties of embryonic stem cells [12]. Generation of hiPSCs and their differentiation into a variety of cells and organoids have allow to set up versatile, non-invasive, ethically sustainable, cruelty-free, and patient-specific models for the investigation of the mechanisms of human diseases, including viral infections and host–pathogen interactions, for the discovery of new drugs, and for the development of personalized therapies [12]. Human iPSC-derived neural cells and brain organoids have been extensively used to investigate the mechanisms of ZIKV and other flavivirus infection and pathogenesis and have been used as platforms to discover therapeutic targets and to test antiviral compounds [13,14,15,16,17,18,19,20,21,22,23]. Initial studies with hiPSCs studies were crucial to provide evidence of the tropism of ZIKV for human cortical neural progenitor cells and to demonstrate that ZIKV infection impairs cell differentiation and induces apoptosis [14,15], supporting the causative role of ZIKV in fetal microcephaly. Human iPSC-derived brain organoids, which are 3D structures mimicking endogenous human brain development and organization, showed that ZIKV preferentially infects radial glia cells as compared to intermediate progenitors or immature neurons, leading to cell death [16]. Activation of TLR3-mediated innate immune response was one of the mechanisms involved ZIKV deleterious effects on neurogenesis [17]. In addition, the ability of viruses to infect and replicate was found to be dependent on cell differentiation status, since stem cells appear to be refractory to viral infection probably due to constitutive high-level expression of interferon-stimulated genes (ISGs), while cell susceptibility to viral infection increases in differentiated cells [24]. Comparisons with other flaviviruses—e.g., DENV—showed that the tropism for neural stem cells was a distinguishing feature of ZIKV [23], but no extensive comparative analyses have been performed with typically neurotropic flaviviruses like WNV.

In this study, we investigated the infectivity, tropism, and replication kinetics of ZIKV, in comparison with WNV and DENV in hiPSCs, hiPSCs-derived neural stem cells (NSCs) and undifferentiated neurons, and the effect of viral infection on host innate antiviral responses. In addition, we exploited hiPSCs to model ZIKV infection in the embryo and during stem cell differentiation towards the neuronal lineage. The results of this study confirmed the tropism of ZIKV for NSCs, but showed that WNV replicated in these cells with much higher efficiency, inducing massive cell death. Although with lower efficiency, all flaviviruses could also infect pluripotent stem cells and neurons, inducing similar patterns of antiviral innate immune response gene expression. While showing the usefulness of hiPSC-based infection models, these findings suggest that additional virus-specific mechanisms, beyond neural tropism, are responsible for the peculiarities of disease phenotype in humans.

## 2. Results

### 2.1. Infection and Replication Kinetics of Flaviviruses on hiPSCs, NSCs, and Neurons

**Figure 1:**
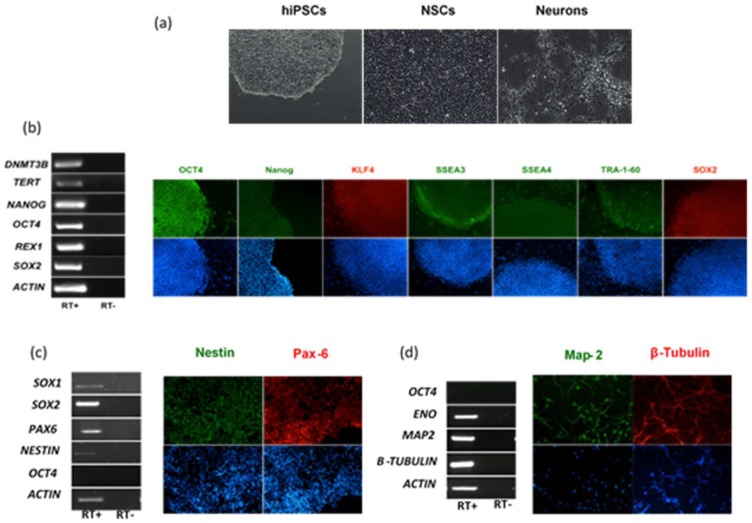
Characterization of human induced pluripotent stem cells (hiPSCs), hiPSC-derived neural stem cells (NSCs) and neurons. (**a**) Representative light microscope images of hiPSCs, NSCs, and neurons used to model flavivirus infection; (**b**) RT-PCR analysis of immunofluorescence (IF) staining of hiPSCs for a panel of pluripotency markers; (**c**) RT-PCR analysis and IF staining of NSCs for the NSC differentiation markers SOX1, SOX2, Nestin, and PAX6; (**d**) RT-PCR analysis and IF staining of neurons for the neuron differentiation markers ENO, MAP-2, and β-Tubulin. The stemness marker OCT4 was not expressed in NSCs and neurons, as expected. Nuclei were stained with DAPI. Magnification 20×.

The different cellular tropism and ability to counteract host cell intrinsic immunity is conceivably one of the key factors that explains the diverse clinical presentation of infections caused by genetically related viruses. This is the case of neurological disease associated with flavivirus infection. In this part of the study, we evaluated and compared the infection and replication efficiency of flaviviruses in pluripotent stem cells and cells differentiated towards the neural lineage. Among flaviviruses, we chose WNV as representative of neurotropic viruses, DENV as representative of viruses associated with hemorrhagic fever, which may cause neurological complications in patients with severe shock syndrome, and ZIKV, which is unique among flaviviruses for its ability to infect the fetus during pregnancy and cause severe developmental defects of the central nervous system. For infection modeling, we used human iPSCs (hiPSCs), which share pluripotency features of human embryonic stem cells, NSCs and immature neurons, which represent the neural progenitor cells and neurons in the developing fetal brain, respectively. hiPSCs clones were generated by reprogramming of erythroblasts expanded from peripheral blood mononuclear cells (PBMCs) with Sendai virus vectors carrying the four Yamanaka factors (Oct4, Sox2, Klf4, and c-Myc). Human NSCs and immature neurons were obtained from the neural differentiation of human iPSC clones (Figure 1).

**Figure 2:**
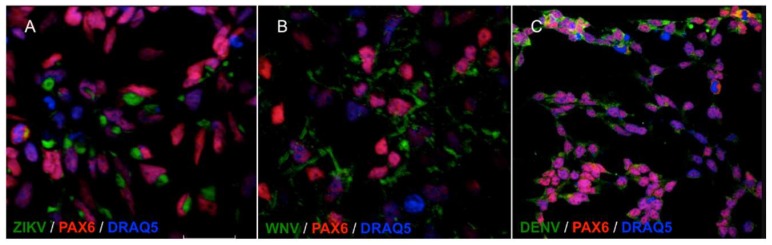
Confocal microscopy images of NSCs infected with ZIKV (**A**), WNV (**B**) and DENV-2 (**C**), MOI 1, 72 hpi. Flavivirus E glycoprotein was stained with goat anti-mouse IgG H&L FITC-conjugated (green); the NSCs marker PAX6 was stained with goat anti-rabbit IgG AlexaFluor546-conjugated (red). Merged images, 60× magnification, zoomed two times.

NSCs were infected with viruses at the multiplicity of infection (MOI) of 1. MOI was defined as the ratio of the number of infectious virus particles measured as plaque forming units, PFU, or tissue culture infectious dose 50%, TCID50, to the number of target cells that were present in a well. Target cell infection was assessed at 72 h post infection (hpi) by immunofluorescence staining with a monoclonal antibody targeting the flavivirus envelope (E) glycoprotein. The percentage of infected cells was estimated by fluorescence microscopy at 40× magnification by counting the number of positive cells to the total number of cells in five fields of view in triplicate experiments. The average infection rate was estimated as 25% ± 8%, 80% ± 12%, and 15% ± 5% of NSCs infected with ZIKV, WNV, and DENV-2, respectively. Representative confocal microscopy images of ZIKV, WNV, and DENV-2-infected NSCs are shown in Figure 2.

**Figure 3:**
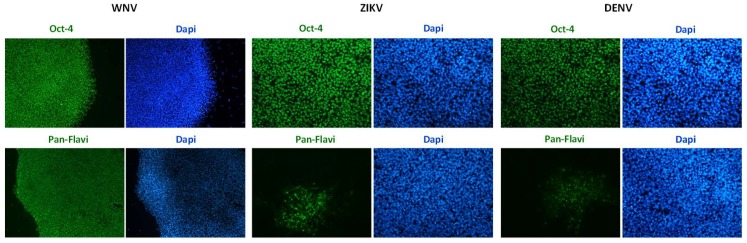
WNV and ZIKV infection of hiPSCs. Immunofluorescence staining of flavivirus envelope E protein and the pluripotency marker OCT4 was performed at 72 hpi with ZIKV and WNV, MOI 1. Nuclei were stained with DAPI; magnification 20×.

**Figure 4:**
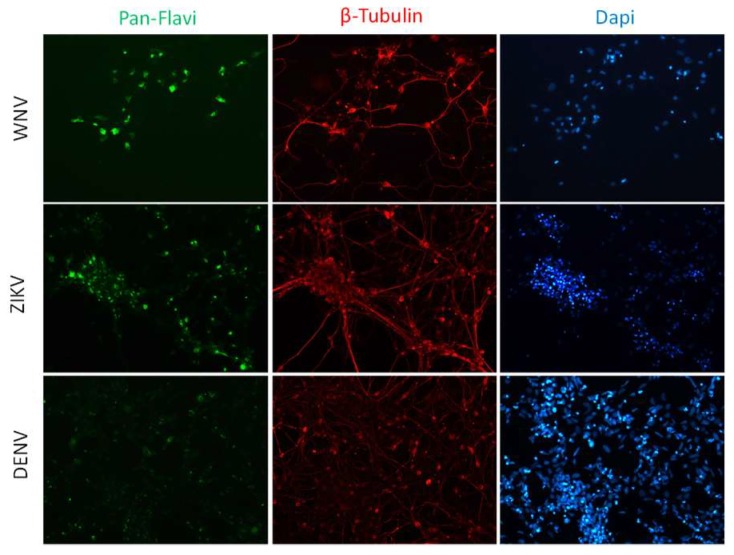
WNV and ZIKV infection of neurons. Immunofluorescence staining of flavivirus E protein (green) and the neuron marker β-tubulin (red) in neurons at 72 hpi with WNV and ZIKV at MOI 1. Nuclei were stained with DAPI; magnification 20×.

Infection efficiency was also evaluated in hiPSCs and in hiPSC-derived neurons (Figure 3 and Figure 4). In both cell types, WNV infection was more efficient than ZIKV and DENV-2 infection. In particular, 30% ± 7%, 80% ± 10%, and 25% ± 5% of hiPSCs and 40% ± 10%, 60% ± 15%, and 30% ± 8% of neurons showed positive anti-E immunostaining after infection with ZIKV, WNV, and DENV-2 at MOI 1, respectively.

**Figure 5:**
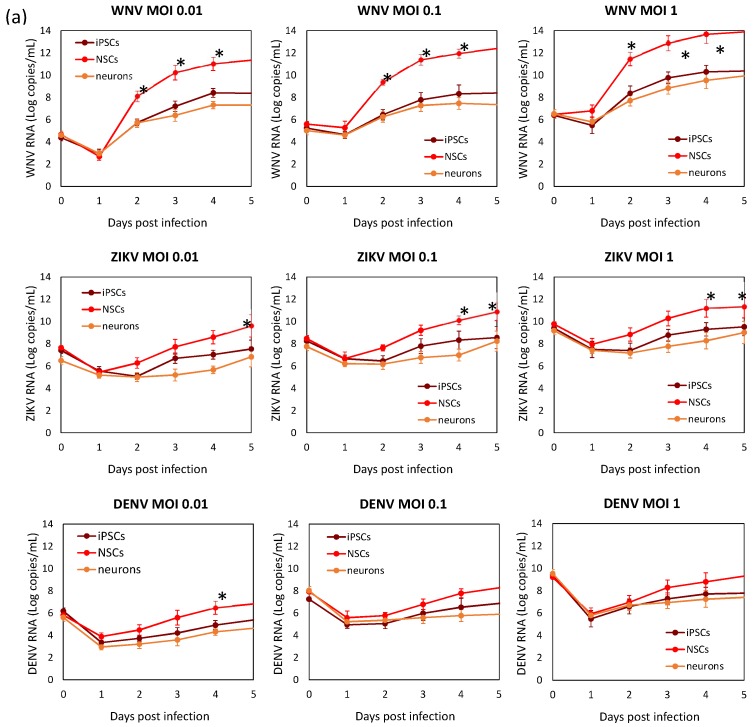

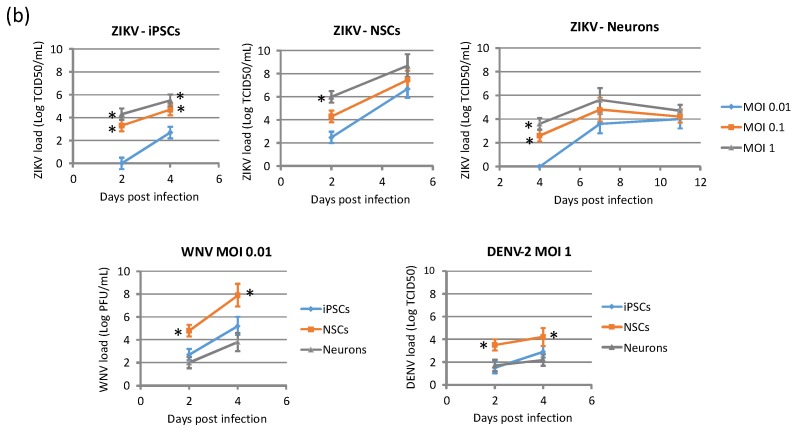
WNV, ZIKV, and DENV replication kinetics of hiPSCs, NSCs, and neurons. Cell were infected with viruses at MOI 0.01, 0.1, and 1. (**a**) Viral RNA load was measured daily in cell culture supernatant by qRT-PCR. (**b**) Infectious viral titer in cell culture supernatant measured by plaque assay or TCID50 assay in Vero cells. qRT-PCR data are shown as the means ± SD from three independent experiments conducted in quintuplicate. Viral load data are shown as the means ± SD from three independent experiments in duplicate. * *p* < 0.05, Student’s *t*-test.

Viral replication kinetics was evaluated in time-course experiments of infection at different MOIs in hiPSCs, NSCs, and neurons (Figure 5). Viral RNA load and infectious virus yield were measured by qRT-PCR and TCID50 assay, respectively, in supernatants of infected cells. Growth curves showed that flavivirus replication was more efficient in NSCs than in hiPSCs and neurons. Comparison of replication kinetics of the different flaviviruses showed that WNV had the highest efficiency, as shown by a more rapid increase in viral titer, followed by ZIKV, while DENV-2 showed the lowest replication efficiency in NSCs.

### 2.2. Cytopatic Effects and Cell Death by Apoptosis in Infected Cells

**Figure 6:**
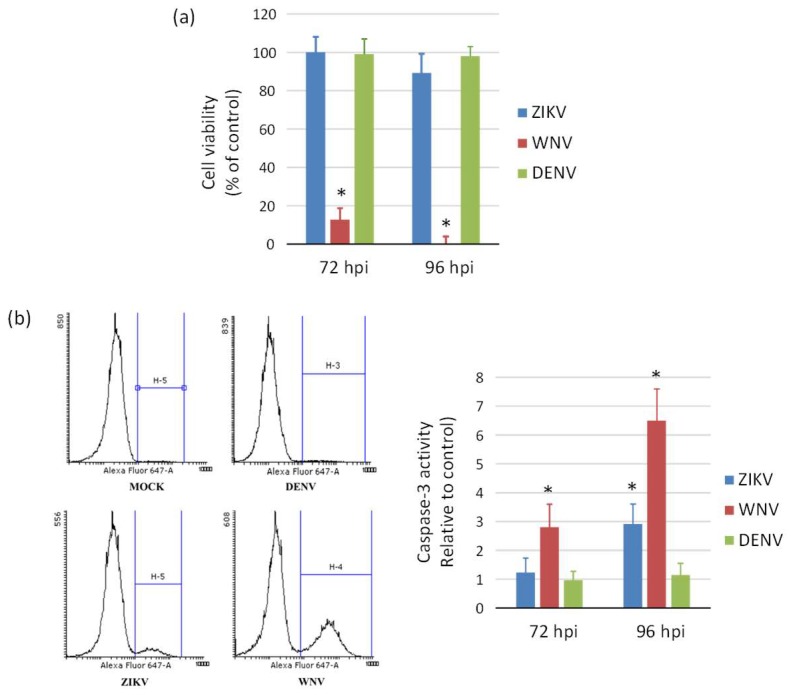
Induction of cell death (**a**) and apoptosis (**b**) in WNV, ZIKV, and DENV infected neural stem cells. (a) Cell viability was measured by MTT assay at 72 h and 96 post infection with ZIKV, WNV, and DENV-2 at MOI 1. (**b**) Caspase-3 activity was measured by flow cytometry in cells harvested at 72 h post infection with ZIKV, WNV, and DENV-2 at MOI 1. Data are shown as the means ± SD from three independent experiments in duplicate. * *p* < 0.05, Student’s *t*-test.

ZIKV exerted a cytopathic effect (CPE) not only in NSCs, but also in hiPSCs and neurons infected at different MOIs. An aggressive CPE was observed also in WNV-infected cells, while no CPE was apparent in DENV-2-infected cells at 96 hpi, in agreement with the lower replication efficiency of this virus. Accordingly, measurement of cell viability by the MTT assay showed massive death of NSCs at 96 hpi with WNV, while no significant decrease in cell viability occurred following infection with ZIKV and DENV-2 (Figure 6a). A significant 3-fold and 6-fold increase of caspase-3 activity, a marker of cell apoptosis, was demonstrated in ZIKV- and WNV-infected NSCs, respectively, compared to mock-infected control NSCs at 96 hpi, while no significant changes in cell apoptosis were measured in DENV-2-infected NSCs (Figure 6b).

### 2.3. Expression of Innate Antiviral Immune Response Genes in Infected Cells

**Figure 7:**
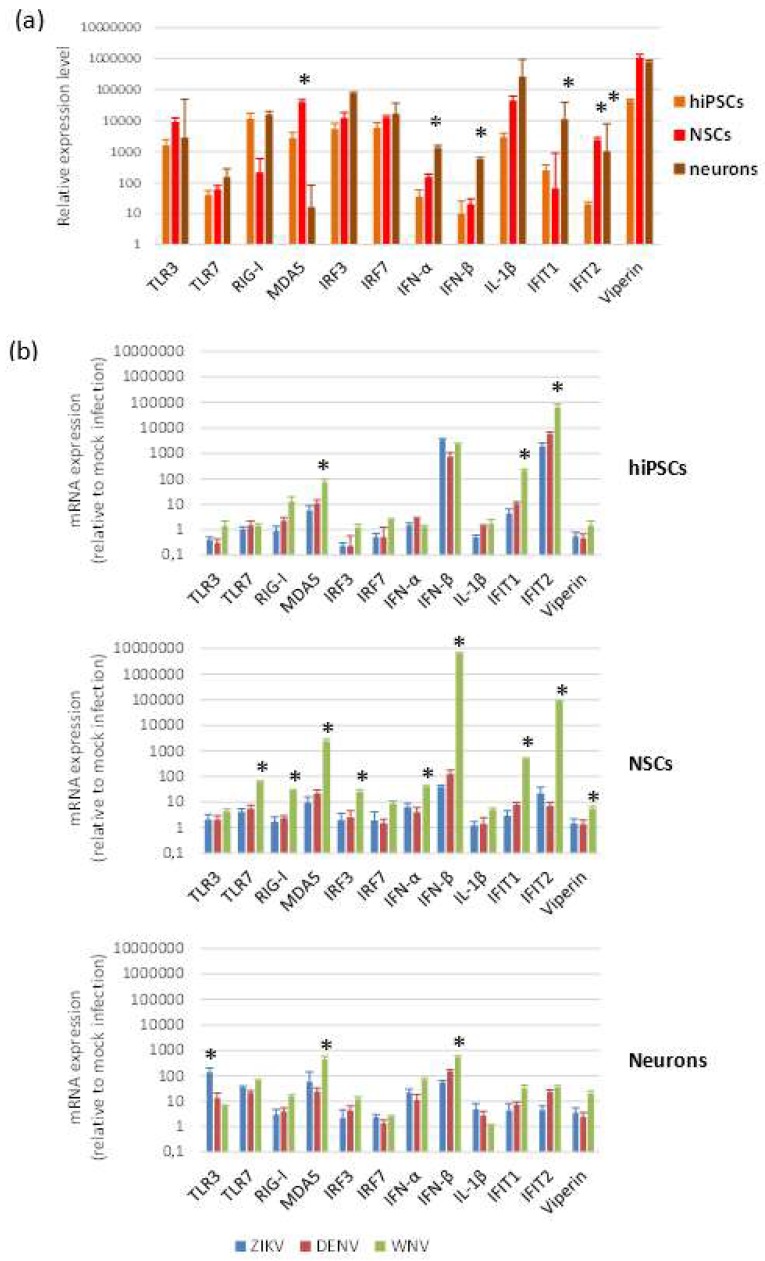
Antiviral innate immune response to flavivirus infection. qRT-PCR analysis of innate antiviral immune response mRNA levels in baseline conditions (**a**) and following flavivirus infection (**b**). hiPSCs, NSCs, and neurons were infected with ZIKV (MOI 1), DENV-2 (MOI 1), and WNV (MOI 0.01) and harvested at 96 hpi. Data are represented as mean ± SD from three independent experiments conducted in quintuplicate. * *p* < 0.05, Student’s *t*-test.

To investigate the innate antiviral response of cells to flavivirus infection, we measured by qRT-PCR the mRNA levels of a panel of genes, which represent key players in host restriction of flavivirus infection. The panel included genes encoding pattern recognition receptors (PRRs) activated by viral dsRNA, i.e., *TLR3, DDX58/RIG-I, IFIH1/MDA5*, and by ssRNA (*TLR7*); genes encoding interferon (IFN) regulatory factors 3 (*IRF3*) and 7 (*IRF7*); interferon type I IFNα (*IFNA1*) and IFNβ (*IFNB1*); IFN-stimulated genes with antiviral activity, i.e., IFN-induced protein with tetratricopeptide repeats 1 (*IFIT1*) and 2 (*IFIT2*) and Viperin (*RSAD2*); the mediator of the inflammatory response IL-1β (*IL1B*). Intrinsic expression of innate antiviral immune response genes, analyzed in cells harvested at different time points after seeding, showed no significant differences, while some variability in mRNA expression levels was observed according to cell differentiation status (Figure 7a).

Modulation of genes involved in innate antiviral immune response was analyzed by qRT-PCR on hiPSCs, NSCs, and neurons after infection with ZIKV (MOI 1), WNV (MOI 0.01), and DENV-2 (MOI 1) at 48 and 96 hpi. While no relevant changes in mRNA levels were apparent at 48 hpi, except *IFIT2* mRNA, which was significantly higher in infected NSCs than in mock-infected control NSCs, induction of expression of most genes was significant at 96 hpi (Figure 7b). In NSCs, but also in hiPSCs and neurons, all flaviviruses significantly up regulated the *MDA5*, *IFNB1*, *IFIT1*, and *IFIT2* genes. WNV, although used at lower MOI than the other flaviviruses (since infection with MOI 1 led rapidly to complete cell death), induced in the highest levels of innate immune response gene transcripts, especially in NSCs, with a particularly strong stimulation of *IFNB1* and the effector *IFIT2*, a critical mediator involved in antiviral immunity.

### 2.4. ZIKV Infection and Replication during Neurogenesis

**Figure 8:**
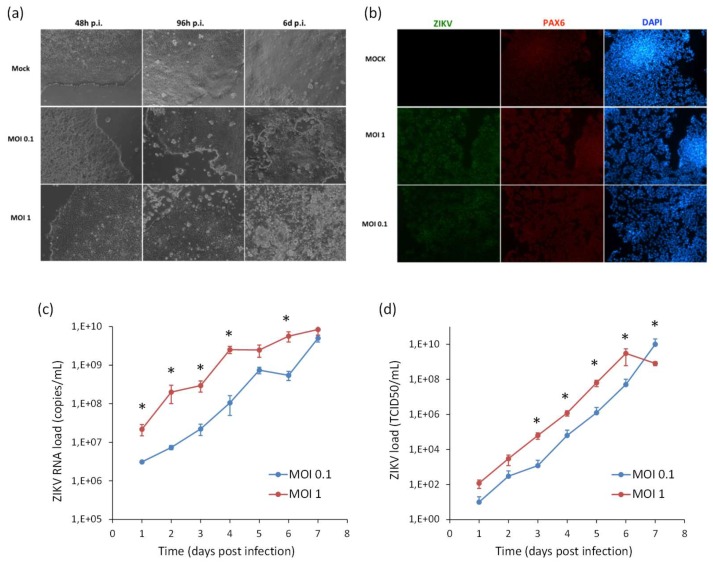
Analysis of ZIKV infection in differentiating hiPSCs. (**a**) Cytopathic effect of ZIKV in differentiating hiPSCs (MOI 1) at 2, 4, and 6 dpi. (**b**) Immunofluorescence staining assessing the expression of flavivirus E protein (green) and Pax6 (red) in ZIKV-infected differentiating hiPSCs (MOI 1) at 7 dpi; nuclei were stained with DAPI; 20× magnification. (**c**) ZIKV load in culture supernatants measured by TCID50 assay from 1 to 7 dpi. qRT-PCR data are shown as the means ± SD from three independent experiments conducted in quintuplicate. Viral load data are shown as the means ± SD from three independent experiments in duplicate. * *p* < 0.05, Student’s *t*-test.

In order to model ZIKV infection during neurogenesis, hiPSCs and NSCs were infected with ZIKV at MOI 0.1 and 1, followed by triggering the differentiation process into NSCs and neurons, respectively. Supernatants were harvested every 24 h until 7 days pi for viral titration by qRT-PCR and TCID50 assay. In hiPSCs infected prior to their differentiation into NSCs, ZIKV replicated efficiently and induced massive cell death. At 7 dpi, when the neural induction was almost completed, most cells were positive for ZIKV E protein. Cells also expressed PAX6, a marker of the early ectoderm, thus indicating that ZIKV infection did not impair the differentiation process (Figure 8).

**Figure 9:**
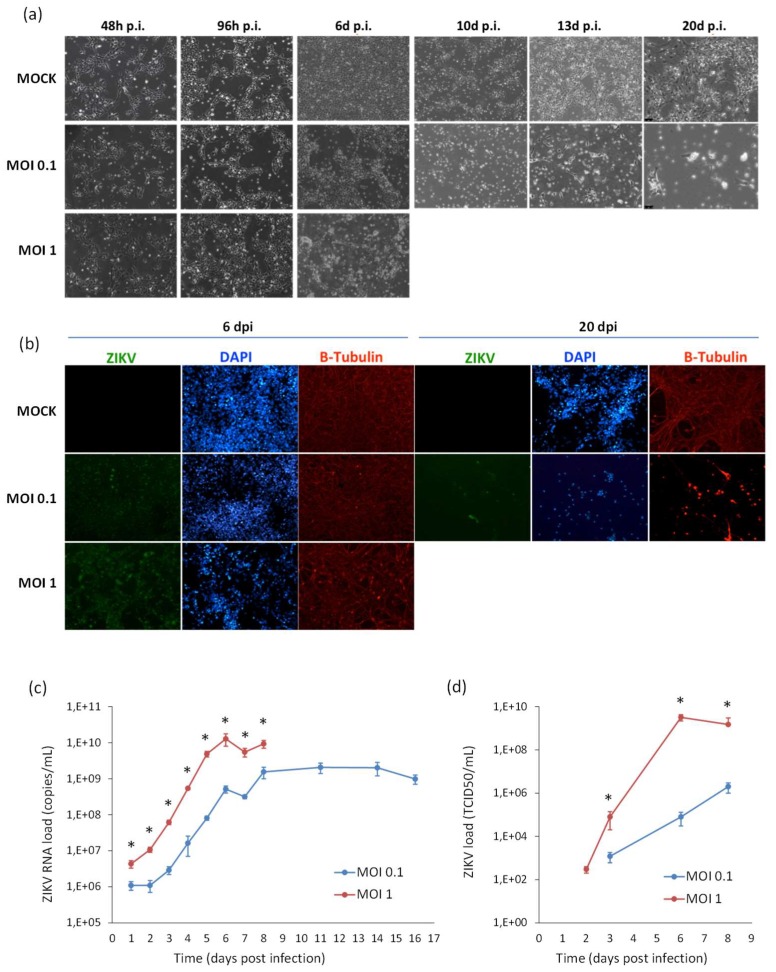
Analysis of ZIKV infection in differentiating NSCs. (**a**) CPE of ZIKV-infected differentiating NSCs at different time-points post infection. (**b**) Immunofluorescence staining assessing the expression of flavivirus E protein (green) and β-Tubulin (red) in ZIKV-infected differentiating NSCs at 6 and 20 dpi. Nuclei were stained with DAPI. 20× magnification. (**c**) ZIKV RNA load measured by qRT-PCR in cell culture supernatants. (**d**) ZIKV load measured by TCID50 assay in cell culture supernatants. qRT-PCR data are shown as the means ± SD from three independent experiments conducted in quintuplicate. Viral load data are shown as the means ± SD from three independent experiments in duplicate. * *p* < 0.05, Student’s *t*-test.

Likewise, ZIKV replicated efficiently in NSCs infected prior to their differentiation into neurons. A progressive increase of ZIKV titer was measured in cell supernatant, followed by a decline due to massive cell death. Also in this case, ZIKV infection did not block the neural differentiation process, as shown by expression of the neuron-specific protein β-Tubulin in infected cells (Figure 9).

### 2.5. ZIKV Infection during Embryogenesis

**Figure 10:**
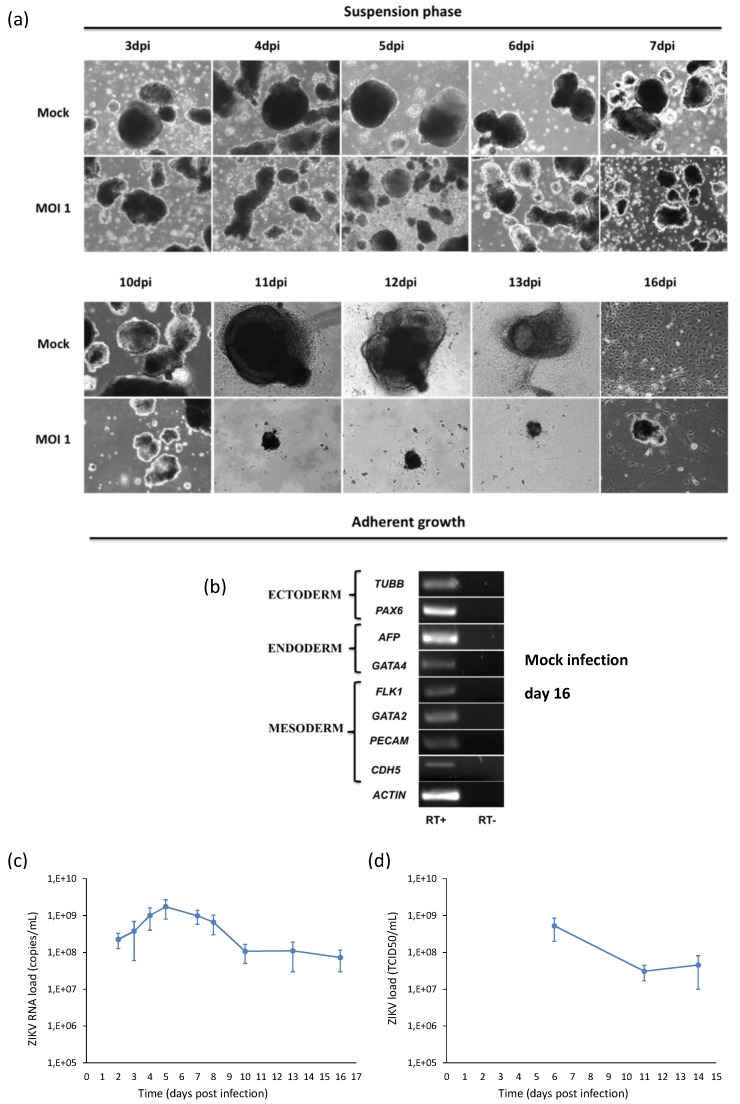
Analysis of ZIKV infection in hiPSCs differentiating into embryoid bodies (EBs). (**a**) hiPSCs were infected with ZIKV at MOI 1 and, after 2 days, cells were detached by collagenase IV treatment in order to initiate differentiation of EBs. EBs were grown in suspension for the first 7 days and in adhesion on gelatin-coated surfaces on the following days. 10× magnification. (**b**) RT-PCR products derived from the amplification of markers belonging to the three germ layers in EBs at day 16. Only the results of mock infection are shown, since the test could not be performed in infected cells because of the poor quantity and quality of purified RNA. (**c**) ZIKV RNA load measured by qRT-PCR and (**d**) infectious ZIKV titer determined by TCID50 assay in the supernatant of ZIKV-infected hiPSCs induced to EB differentiation (MOI 1).

Modeling of ZIKV infection during embryogenesis was performed by infection of hiPSCs with ZIKV MOI 1 during their differentiation into embryoid bodies (EBs), i.e., micro-tissue similar to native tissue elements. Viral infection was monitored by collecting the supernatants of infected cells and analyzing viral RNA copy number by qRT-PCR at different time points pi. Viral RNA load increased with time, reached a peak at 5 dpi and then decreased in parallel with the decrease of the number of live cells (Figure 10a). As shown in the Figure 10b, ZIKV infection of hiPSCs and subsequent random differentiation into EBs growing in suspension outlined a progressive reduction in the volume and number of the EBs that displayed jagged edges when compared to mock controls. When the adhesion stimuli were added, infected EBs were not able to fully attach to the gelatin-coated plastic. Surviving adherent infected cells started to die with subsequent detachment.

## 3. Discussion

In the epidemiological context of the recent ZIKV epidemics in Asia and the Americas, the continuous expansion of WNV in Europe, and the dramatic increase of dengue epidemics, the aim of this study was to set up in vitro models of viral infection based on hiPSC-derived neural cells and to use these models to investigate and compare interaction of the three viruses with host human cells.

Although hiPSC-derived NSCs, neurons and brain organoids have been extensively used to investigate ZIKV neural tropism and pathogenesis in comparison with other flaviviruses [14,15,16,17,19,21,23,24], a novelty of this study is the detailed investigation and comparison of the effects of different flaviviruses on hiPSCs, NSCs, and neurons. Three representative flaviviruses associated with human disease—i.e., ZIKV, WNV, and DENV—were chosen for experiments.

In agreement with the accumulating evidences reporting infection of NSCs by ZIKV [14,15,16,17], this study confirmed the permissiveness of hiPSCs-derived NSCs to productive ZIKV infection. Immature neurons and hiPSCs also supported ZIKV replication, although at lower efficiency. These data are in contrast to a previous study showing that infection of undifferentiated hiPSCs and immature neurons was inefficient [14]. Different observations were reported in other studies that demonstrated permissiveness of developing immature neurons to ZIKV infection as well as relative resistance of mature neurons in adult human brains [25].

DENV-2 and WNV also infected NSCs, hiPSCs, and neurons. DENV-2 infected hNSCs but at lower rate than ZIKV, in agreement with previous reports [17]. At variance, WNV infected and replicated in all cell types with significantly higher efficiency than ZIKV and DENV-2. Notably, WNV was able to infect hiPSCs and immature neurons, although at lower rate than NSCs. Studies in the literature report resistance of undifferentiated embryonic stem cells and hiPSCs to WNV infection [26] and increase of permissiveness to viral infection as cells progress towards neural differentiation [26,27]. The high resistance of stem cells, including hiPSCs, to viral infection was ascribed to the intrinsic expression of a set of ISGs with antiviral activity [24]. Likewise, differential expression of innate immune programs in neuronal subtypes in the cerebral cortex confer differential susceptibility to WNV infection and other neurotropic viruses [28].

Both ZIKV and WNV exerted CPEs in hiPSCs, NSCs and neurons, leading to massive cell death, due in part to apoptosis mediated by activation of caspase-3, in agreement with previous reports [14,29]. At variance, DENV-2 infection had milder CPEs on NSCs and did not induce cell apoptosis, confirming results in literature [17]. Cell viability assays demonstrated that WNV induced massive cell death with complete cell loss at 96 hpi, while only 10% reduction of NSC viability was observed after ZIKV infection. These findings suggest that ZIKV replication is slower and the virus can persist for a longer time in infected cells.

Regarding the investigation of the innate antiviral response during ZIKV infection, studies based on different cell types and viral strains led to different observations. In primary human skin fibroblasts, ZIKV Asian lineage induced the upregulation of three important PRRs known to be activated by viral dsRNA, specifically TLR3, RIG-I, and MDA5, and, also, other factors involved in downstream pathways, such as IRF7, IFNα, IFNβ, and C-C motif chemokine ligand 5 (CCL5) [30]. In the human lung epithelial cell line A549, ZIKV (H/PF/2013) stimulated the production of type-I IFN, ISGs and pro-inflammatory cytokines, in particular IL-1β [31]. In human fetal neural progenitors, ZIKV Asian lineage induced partial CPE and did not stimulate cytokine secretion [32]. At variance, Dang et al. [17] showed that TLR3 was upregulated in cerebral organoids and human neurospheres after ZIKV MR766 infection, and TLR3 activation was associated with cell apoptosis, organoid shrinkage, and dysregulation of neurogenesis induced by ZIKV infection.

In this study, we investigated expression of a set of genes involved in antiviral innate immunity and showed that ZIKV, WNV, and DENV-2 infection induced a similar pattern of antiviral response in NSCs, neurons and hiPSCs, characterized by a marked upregulation of the dsRNA sensor MDA5 and the IFN-induced factors with antiviral activity IFIT1 and IFIT2. The highest expression levels of the antiviral genes were observed in cells infected by WNV, while ZIKV and DENV infections had milder effects on host innate antiviral response. These differences could be ascribed to the higher burden of WNV-infected cells due to higher infection and replication efficiency of the virus but also to the extent of viral inhibition of host innate immune response.

Previous studies demonstrated an antiviral function of IFIT2 in controlling viral replication in the CNS [33], and IFIT2 has been suggested to have antiviral activity against WNV [34]. However, WNV uses the 2′ O-methylation of the 5′ guanosine cap as a mechanism to evade the antiviral effects of IFIT1 and IFIT2 [35]. This is consistent with our observations of a stronger upregulation of IFIT2 following WNV infection than ZIKV and DENV-2 infection. Also, IFIT2 triggers a mitochondrial pathway of apoptosis accompanied by the pro-apoptotic proteins Bax and Bak with the activation of caspase-3 [36]. Accordingly, in our study we could observe overexpression of *IFIT2* in infected NSCs and activation of caspase-3.

The literature reports absent or low levels of innate immune response against viral infection in mESCs [37], hESCs [38], and also in hiPSCs [37], which are characterized by intrinsic high level expression of a set of genes with antiviral activity which confer resistance to viral infection [24]. At variance, our data demonstrate that hiPSCs infected by flaviviruses were able to activate an innate antiviral response characterized by upregulation of *MDA5*, *IFIT1*, *IFIT2*, and other ISGs. Therefore, innate immunity represents an uncharacterized property of pluripotent stem cells that requires further studies to elucidate the molecular mechanisms.

Recent reports demonstrated that ZIKV directly infects NSCs of the fetus and impairs growth in mice [39]. ZIKV has a negative impact on neurogenesis either by abrogating it completely or by disrupting neural cell organization [17]. To further investigate if ZIKV could impair the formation of neural precursors and neurons, the effect of viral infection during the neural differentiation process, which is critical for brain development, was analyzed. Our experiments showed that ZIKV infected and replicated in hiPSCs during their differentiation into NSCs, inducing CPE and cell death, but it did not affect the differentiation process, as demonstrated by the maintenance of the NSC marker Pax6 at the end of the neural induction phase. Likewise, in NSCs infected prior to their differentiation into neurons, ZIKV did not block neuronal development, as confirmed by the expression of the neuron marker β-tubulin during neurogenesis. In these cells, ZIKV induced massive cell death that increased with time, leading to a complete disruption of cell monolayer, so the neurogenesis process could not be completed. Similar findings were reported with human NSCs growing as neurospheres and brain organoids [17]. Since hiPSCs and NSCs do not present organization features typical of 3D structures, we used EBs to investigate the effect of ZIKV infection during embryogenesis. Cells forming EBs exhibit heterogeneous patterns of differentiated cell types from the three germ lineages and are able to respond to similar cues that direct embryonic development [40]. Therefore, this 3D structure enables differentiation and morphogenesis yielding micro-tissues similar to native tissue structures. ZIKV infected EBs leading to a reduction in their volume and number. Consequently, the surviving EBs were not able to fully attach to the substrate and died. These data suggest that ZIKV induces cell death and abrogates cell differentiation and EBs formation, which can explain the impairment of embryo development observed after ZIKV infection in vivo.

In conclusion, this study highlighted similarities and differences in ZIKV, WNV, and DENV mechanisms of infection of pluripotent stem cells and neural cells and induction of cell damage and antiviral response, which warrant further investigation. Several factors, both viral and cellular, are involved in the difference in infectivity, replication kinetics, and pattern of host antiviral response. These factors include, for example, expression levels of entry molecules on host cells and tissues and biding affinity with viral E proteins [41,42]. Other mechanisms are the specific interaction of viral nonstructural proteins with host targets, such as in the case of the secreted NS1 protein, which modulates endothelial barrier function in a tissue-specific manner for the different flaviviruses [43]. Flaviviruses also differ in their ability to counteract host innate immunity through nonstructural proteins and in their sensitivity to antiviral response [44,45].

## 4. Materials and Methods

### 4.1. Cells and Culture Protocols

Human iPSCs were generated by reprogramming of erythroblasts from healthy donors using Sendai virus-based vectors, as reported [46]. Blood samples were collected from donors after they gave informed consent. The research involving human samples was conducted in accordance with the Declaration of Helsinki principles and with the Review Board and Ethics Committee of Padova University Hospital. Briefly, for hiPSC reprogramming, PBMCs were separated from whole blood samples, expanded and differentiated for 10 days in EM medium to obtain the erythroblast population. Then, 2 × 10^5^ cells were transduced with four Sendai virus vectors expressing the Oct4, Sox2, Klf4, and c-Myc, respectively, at a MOI of 10. Clones derived from reprogramming were manually picked, expanded, and stored in liquid nitrogen. Human iPSC clones were grown on irradiated mouse embryonic fibroblast (MEF, Global Stem, Thermo Fisher Scientific, Waltham, MA, USA) feeder layer with hiPSCs medium containing Dulbecco’s modified Eagle’s medium Nutrient Mixture F-12 (DMEM/F12), 20% Knockout serum replacement (KSR), 1% non-essential amino acids (NEAA), 1% GlutaMAX 100×, 1% Penicillin/Streptomycin, 1% of β-mercaptoethanol 1000× (all from Thermo Fisher Scientific) and basic fibroblast growth factor (bFGF, ORF Genetic, Kópavogur, Iceland) 10 ng/mL; or onto feeder-free layer Geltrex substrate (Thermo Fisher Scientific) with serum-free StemMACS medium (Miltenyi Biotec, Bergisch Gladbach, Germany) supplemented with 1% penicillin/streptomycin (Thermo Fisher Scientific).

Neural induction of hiPSCs was performed using Gibco PSC Neural Induction medium (Thermo Fisher Scientific) containing neurobasal medium and neural induction supplement 50×. Neural induction medium was changed every other day from day 0 to day 7 of neural induction. From day 7, primitive NSCs were maintained in NSCs expansion medium containing 50% neurobasal medium, 50% Advanced DMEM/F12 and neural induction supplement 50×, which was changed every other day.

Expanded NSCs were differentiated into immature neurons. NSCs were seeded onto polyornithine- (20 µg/mL) and laminin- (10 µg/mL) (all from Sigma-Aldrich, St. Louis, MO, USA) coated culture dishes in NSCs medium. The next day, NSCs medium was replaced with neural differentiation medium consisting of Neurobasal medium, 2% B-27 Supplement, GlutaMAX 100× 2mM and 1% penicillin/streptomycin (all from Thermo Fisher Scientific). The culture medium was changed every 3–4 days.

Embryoid bodies (EBs) formation was performed by plating hiPSCs in ultra-low-density attachment plates (Corning Incorporated, Corning, NY, USA) in hiPSCs medium without bFGF. The medium was changed every 3 days. After 7 days, EBs were seeded on 0.1% gelatine- (Merck Millipore, Burlington, MA, USA) coated 6-well tissue culture plate with DMEM, 10% FBS, 1% penicillin/streptomycin, 2 mM GlutaMAX (Thermo Fisher Scientific) and the medium was changed every 3 days. After 7 days of growth adhesion, cells were harvested using Trypsin enzyme (Thermo Fisher Scientific).

### 4.2. Embryoid Bodies (EBs) Test

hiPSC clones growing on MEFs were detached from the well with collagenase IV (Thermo Fisher Scientific) and plated in ultra-low-attachment plates (Corning Incorporated), in iPS medium without b-FGF. The medium was changed every three days; after seven days, EBs were transferred into a 0.1% gelatin pre-coated 6-well plate with DMEM 10% FBS 1% penicillin–streptomycin, 2 mM Glutamax (all from Thermo Fisher Scientific).

After a week of growth in adhesion, cells were harvested by trypsinization and total RNA was collected and reverse-transcribed into cDNA. Quantitative RT-PCR analysis was performed to assess the expression of the three germ layers markers: ectoderm (*TUBB*, *PAX6*), mesoderm (*PECAM, CDH5*, *GATA2*, *FLK1*), and endoderm (*AFP*, *GATA4*).

To model ZIKV infection during embryogenesis, ZIKV infection was performed during EB formation. Specifically, hiPSCs were infected at MOI 1 and, after 2 days, cells were detached by collagenase IV treatment in order to initiate differentiation of EBs. Detached infected hiPSC colonies were suspended in iPSC medium without bFGF supplementation to prevent stemness retention, and grown in suspension for 7 days. At day 7, growing EBs were plated on 0.1% porcine gelatin in order to allow them to grow in adhesion. Viral infection was monitored by viral titration in the supernatants of infected cells.

### 4.3. Viral Strains and Infections

The following viral strains were used in experiments: ZIKV strain H/PF/2013 (GenBank KJ776791), Asian lineage, clinical isolate from French Polynesia, 2013; WNV strain AUT/2008 (GenBank KF179640), lineage 2, goshwak isolate from Austria, 2008; DENV serotype 2 strain, clinical isolate. Viruses were grown on Vero cells to generate viral stocks at titer of 1 × 10^6^ TCID50/mL (ZIKV), 1.7 × 10^7^ pfu/mL (WNV), and 2 × 10^5^ TCID50/mL (DENV-2).

### 4.4. Infections with ZIKV, WNV, and DENV-2

Cells were seeded in tissue culture well plates; after incubation at 37 °C and 5% CO_2_ for 24 h, cell growth medium was removed and replaced with infection medium, containing the virus diluted in DMEM with 1% penicillin/streptomycin (Thermo Fisher Scientific) at the specified multiplicity of infection (MOI). After incubation at 37 °C and 5% CO_2_ for 1 h and 30 min, the infection medium was replaced with growth medium and cells were maintained at 37 °C and 5% CO_2_. Mock-infected Vero cells were used as negative control in all infection experiments.

### 4.5. Analysis of Virus Replication Kinetics

Viral replication kinetics was measured in cell culture supernatants and cell lysates collected at different time points pi. by using quantitative qRT-PCR with primers and TaqMan-probe sets specific for ZIKV NS5, WNV NS5, and DENV NS5, plaque count assay, and TCID50 assay.

### 4.6. Analysis of Gene Expression by RT-PCR and qRT-PCR

RT-PCR was performed to evaluate the expression of the pluripotency genes (*OCT4*, *NANOG*, *DNMT3B*, *TERT*, *REX1*, and *SOX2*); the differentiation genes of the three germ layers (*TUBB, PAX6* (ectoderm), *PECAM, CDH5, GATA2, FLK1* (mesoderm), *AFP, GATA4* (endoderm)); the neural stem cell-specific genes *NESTIN, PAX6, SOX1, SOX2* and the neuron-specific genes *TUBB, MAP-2, NSE*.

Expression of genes encoding key factors involved in antiviral innate immunity (i.e., *IFIT1*, *IFIT2*, *MDA5, RIG-I, TLR3, TLR7, IL-1β, IRF3, IRF7, Viperin, IFN-α*, and *IFN-β*) was analyzed by qRT-PCR analysis. Nucleic acids were purified from cells by using RNeasy Mini Kit (Qiagen, Hilden, Germany). qRT-PCR was run on the 7900HT Fast Real-Time PCR System instrument (Thermo Fisher Scientific). qRT-PCR results were normalized to GAPDH mRNA and analyzed using the ΔΔCt method.

### 4.7. Immunofluorescence Assays

Cells were fixed in 4% paraformaldehyde solution (PFA; Sigma-Aldrich) in phosphate-buffered saline (PBS, Thermo Fisher Scientific), permeabilized with PBS/0.1% Triton X-100 (Sigma-Aldrich) and blocked in 4% bovine serum albumin (Sigma-Aldrich) in PBS. Then, cells were incubated with the primary antibodies diluted in PBS with 4% BSA. Primary antibodies specific for flavivirus E protein (1:500, Merck Millipore), PAX6 (1:100, Sigma-Aldrich), Nestin (1:100, Abcam, Cambridge, UK), β-Tubulin (1:1000, Abcam), MAP-2 (1:250, Abcam), OCT4 (1:200, Santa Cruz Biotechnology), SSEA3 and SSEA4 (1:50, Abcam), KLF4 (1:50, Santa Cruz Biotechnology), SOX2 (1:50, Merck Millipore) were used. All the antibodies were incubated overnight at 4 °C. The following secondary antibodies, diluted 1:250, were used: anti-mouse IgG FITC (Chemicon international, USA); anti-rabbit IgG Alexa Fluor-546 (Invitrogen, Thermo Fisher Scientific). Nuclei were stained with DAPI (Invitrogen, Thermo Fisher Scientific) or DRAQ5 fluorescent probe solution (Thermo Fisher Scientific). Cells were visualized under a fluorescent microscope (Leica, Wetzlar, Germany) with 20× magnification or a confocal microscope (Leica) with 63x magnification.

### 4.8. Apoptosis Assay

The activity of Caspase 3 was measured at 72 hpi and 96 hpi in mock and infected NSCs plated in quadruplicate in 12 wells plates (7 × 10^4^ cells/well). For each virus a MOI of 1 was adopted. Briefly, cells were detached from the well, fixed with 4% PFA (Sigma-Aldrich, USA) in PBS (Thermo Fisher Scientific), permeabilized in 90% methanol and stored at −20 °C overnight.

The next day, cells were incubated at room temperature with anti-cleaved Caspase-3 primary antibody (Cell Signaling Technology, Danvers, MA, USA) diluted 1:100 in incubation buffer. After 1 h the antibody was removed and the cells were suspended in PBS for data acquisition with Becton Dickinson LSR II Flow Cytometer (BD Bioscience) and analysis using Flowing software.

### 4.9. Cell Viability Assay

Cell survival was evaluated by MTT assay in mock and infected NSCs plated in 96 well tissue culture plates (8 × 10^3^ cells/well). Upon viral infection with a MOI of 1, cell viability was analyzed at 72 h and 96 hpi. Freshly dissolved solution of MTT (5 mg/mL, AppliChem, Darmstadt, Germany) in PBS was added to each well and incubated for 4 h at 37 °C. Then, a solubilization solution (10% sodium dodecyl sulfate and 0.01 M HCl) was added and, after overnight incubation at 37 °C, absorbance was read at 620 nm.

### 4.10. Statistical Analysis

Data were presented as mean value ± standard deviation. Statistical analysis was conducted using an unpaired Student’s *t*-test and the statistical significance was defined as *p* < 0.05.
